# PReS-FINAL-2299: Novel biomarkers for the assessment of pediatric systemic lupus erythematosus nephritis (preliminary report)

**DOI:** 10.1186/1546-0096-11-S2-P289

**Published:** 2013-12-05

**Authors:** A Koutsonikoli, M Trachana, V Tzimouli, E Farmaki, N Printza, A Garyfallos, V Galanopoulou, P Pratsidou-Gertsi, F Papachristou, F Kanakoudi-Tsakalidou

**Affiliations:** 1 1st Department of Pediatrics, Aristotle University of Thessaloniki, "Hippocration" General Hospital, Thessaloniki, Greece; 2 4th Department of Internal Medicine, Aristotle University of Thessaloniki, "Hippocration" General Hospital, Thessaloniki, Greece; 3 Department of Rheumatology, "Papageorgiou" General Hospital, Thessaloniki, Greece

## Introduction

Nephritis is the most common severe manifestation of pediatric Systemic Lupus Erythematosus (pSLE) and the major predictor of poor prognosis. Research for the detection of novel and accurate biomarkers, predictive of the nephritis outcome, is globally in progress. Ethnicity affects the disease outcome but data on purely Caucasian populations are still limited.

## Objectives

The exploration of the role of serum biomarkers, namely anti-nucleosome antibodies (anti-NCS), anti-C1q antibodies (anti-C1q), anti-glomerular basement membrane antibodies (anti-GBM) and high mobility group box 1 protein (HMGB1) in pSLE nephritis and the association of their levels with pSLE nephritis and pSLE disease activity.

## Methods

Twenty-four patients (16 female) with pSLE nephritis (44 serum samples, 22 in active nephritis) and 21 patients (18 female) with pSLE without nephritis (32 serum samples, 19 in active pSLE) were enrolled in the study. The disease control group included 17 patients with nephritis of other causality (Henoch-Schönlein purpura nephritis, IgA nephropathy, postinfectious glomerulonephritis, membranous glomerulonephritis), who provided equal serum samples. The SLICC renal activity score was applied for assessing pSLE nephritis disease activity and ECLAM for global pSLE disease activity. The biomarkers' levels were determined by ELISA.

## Results

The pSLE nephritis patients had significantly higher serum levels of anti-NCS [median: 48.89 (IQR: 31.48-80.81) U/ml versus 12.5 (11.5-27.8) U/ml, p < 0.001], anti-C1q [22.75 (12.77-56.4) U/ml versus 12.5 (12.5-12.5) U/ml, p < 0.001], anti-GBM [3.88 (2.25-6.94) U/ml versus 2.2 (2.2-2.4) U/ml, p = 0.002] and HMGB1 [9.9 (5.7-32.23) ng/ml versus 2.5 (2.5-2.5) ng/ml, p < 0,001], than the patients with nephritis of other causality. Serum anti-GBM levels were significantly higher in the pSLE nephritis patients compared to the pSLE patients without nephritis [3.88 (2.25-6.94) U/ml versus 2.25 (2.2-2.83) U/ml, p = 0.014], while this was not true for the rest of the biomarkers. In the pSLE nephritis patients no correlation was found between serum anti-GBM levels and pSLE nephritis disease activity. Serum anti-NCS and anti-C1q levels were positively correlated with the ECLAM score in the pSLE patients as a whole (p = 0.002, rho = 0.492 and p = 0.007, rho = 0.461, respectively).

## Conclusion

In this pure Caucasian Northern Greek pSLE population, high serum anti-GBM levels were found to be associated with the presence of nephritis, but not with the nephritis disease activity. Serum anti-GBM, anti-NCS, anti-C1q and HMGB1 may be used to differentiate patients with pSLE nephritis from patients with nephritis of other causality. Furthermore, serum anti-NCS and anti-C1q may be useful for the estimation of pSLE disease activity.

## Disclosure of interest

None declared.

